# High-Flow Bypass for Ruptured Aneurysms at Non-branching Sites of the Middle Cerebral Artery: A Case Report

**DOI:** 10.7759/cureus.35903

**Published:** 2023-03-08

**Authors:** Aoto Shibata, Taro Yanagawa, Shin Sugasawa, Syunsuke Ikeda, Toshiki Ikeda

**Affiliations:** 1 Stroke Center, Sagamihara Kyodo Hospital, Sagamihara, JPN

**Keywords:** high-flow bypass, coil embolization, middle cerebral artery, pseudoaneurysm, non-branching sites

## Abstract

Small cerebral aneurysms that occur at non-branching sites are generally considered to have extremely weak aneurysm walls or a pseudoaneurysm formed by a thrombus. Since conventional clipping and coil embolization are difficult and high-risk, trapping with bypass has been considered the preferred treatment method. The aim of this study is to investigate a case of trapping with high-flow bypass for a ruptured aneurysm at non-branching sites of the middle cerebral artery (MCA). In this study, the CT results indicated subarachnoid hemorrhage, while the CT angiography (CTA) results showed a small aneurysm at the non-branching site of the MCA M1 segment. Moreover, the intraoperative digital subtraction angiography (DSA) results strongly suggested a pseudoaneurysm. The aneurysm was judged to be a pseudoaneurysm over the rupture site of the true aneurysm sac. Coil embolization was performed, but the treatment was interrupted as the aneurysm completely disappeared during the procedure. However, based on the magnetic resonance angiography findings, the aneurysm reappeared on day five and became enlarged. Thus, trapping with high-flow bypass was performed on day 15 and the patient was cured. Owing to the unusual and noteworthy course of this case, trapping with high-flow bypass was considered to be the safest and most reliable first-choice treatment procedure for pseudoaneurysm at non-branching sites of the MCA.

## Introduction

Small cerebral aneurysms that occur at non-branching sites are typified as an aneurysm of the anterior wall of the internal carotid artery (ICA), and the aneurysm wall is extremely fragile, or has the form of a pseudoaneurysm caused by a thrombus [1]. Owing to the nature of the disease, conventional clipping and coil embolization are difficult and high-risk, and therefore trapping with bypass has been considered the preferred treatment method [2]. However, in recent years, flow diverters (FD) have been widely used in the chronic phase of aneurysm treatment [3], and the etiology of the aneurysm and the best treatment strategy are yet to be determined. In this study, we discuss our experience of encountering a patient with a ruptured aneurysm at non-branching sites of the middle cerebral artery (MCA). We describe its etiology and treatment based on the specific imaging findings and clinical course of the aneurysm.

## Case presentation

History

**Figure 1:**
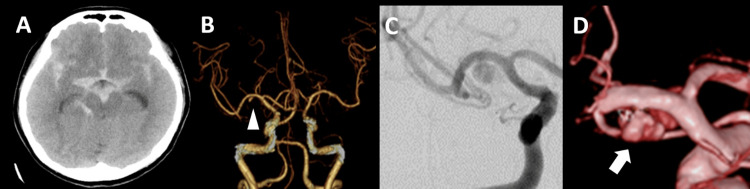
Findings on admission (A) CT upon admission showing diffuse subarachnoid hemorrhage. (B) CT angiography showed a small aneurysm in the middle cerebral artery M1 (arrowhead). (C) DSA showing delayed contrast and congestion findings suspicious for a pseudoaneurysm. (D) Three-dimensional DSA showing a pseudoaneurysm contiguous with a saccular aneurysm at the non-branching segment in the middle cerebral artery M1 (white arrow) CT: computed tomography; DSA: digital subtraction angiography; M1: middle cerebral artery M1 segment

The patient was a 58-year-old woman admitted to the hospital due to a sudden headache and loss of consciousness. Upon admission, she presented with slurred consciousness based on the Glasgow Coma Scale E3V4M5, showing no neurological deficits. The head CT indicated subarachnoid hemorrhage (SAH) (Figure 1A), while the CT angiography (CTA) showed a small 2-mm aneurysm in the M1 segment of the right MCA (Figure 1B). Although the aneurysm was non-branching and small in size, suggesting a dissecting lesion, it was saccular in shape and could have been an aneurysm at the bifurcation with a perforating branch. Thus, digital subtraction angiography (DSA) was performed under general anesthesia with the assumption of embolization. DSA showed delayed contrast and congestion findings that suggested a pseudoaneurysm with an irregular wall and a maximum diameter of 8 mm, which was suspected to have undergone a significant shape change in a short period of time, with a 2.8-mm maximum diameter aneurysm that was visualized at the non-branching segment of the right M1 (Figures 1C, 1D). We speculated that the aneurysm was a pseudoaneurysm over the rupture site of the true aneurysm sac and decided to perform coiling only on the true aneurysm component to prevent rebleeding in the acute phase.

Endovascular procedure

**Figure 2:**
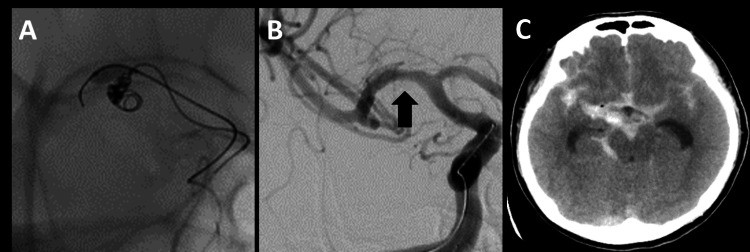
Endovascular intraoperative DSA and postoperative CT findings (A) Balloon-assisted coil embolization was performed. (B) DSA shows that the aneurysm has completely disappeared during the procedure (black arrow). (C) Postoperative CT showed no increased bleeding CT: computed tomography; DSA: digital subtraction angiography

A 6 Fr guiding sheath catheter (Axcelguide, Medikit, Tokyo, Japan) was placed in the right ICA via the trans-femoral approach; a 3.4 Fr intermediate catheter (TACTICS, Technorat Corporation, Aichi, Japan) was guided to the siphon of the ICA, and a microcatheter (Headway 17, MicroVention, Inc., Aliso Viejo, CA) was carefully positioned at the base of the aneurysm. For the first coil, a SMART COIL complex extra soft 2.5 mm x 6 cm (Penumbra, Alameda, CA), which is smaller than the diameter of the true component of the aneurysm, was used. Balloon-assisted embolization was started using SHOURYU HR 4-7 (Kaneka Medix, Osaka, Japan) (Figure 2A). However, the coil dynamics were distorted during the framing of the aneurysm sac, which was judged to be a true component, and embolization was difficult; hence, the coil was temporarily retrieved. Immediately after that, the aneurysm was found to have completely disappeared based on the confirmatory imaging findings (Figure 2B), and the treatment was forced to be discontinued. Postoperative CT showed no evidence of hemorrhage expansion (Figure 2C).

Clinical course

**Figure 3:**
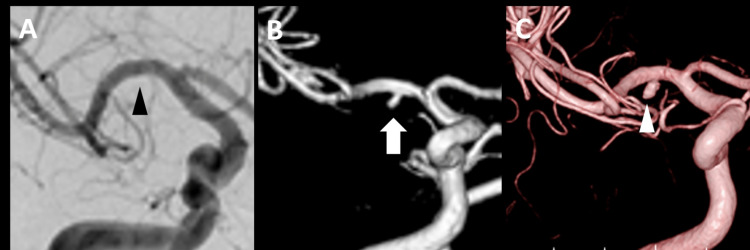
Follow-up DSA and MRA findings (A) DSA on day 2 showing no reappearance of the aneurysm (black arrowhead). (B) MRA on day 5 showed the reappearance of the aneurysm (white arrow). (C) Three-dimensional DSA on day 14 showing an enlarged aneurysm (white arrowhead) DSA: digital subtraction angiography; MRA: magnetic resonance angiography

Treatment for cerebral vasospasm, such as antiplatelet therapy or fasudil, was not administered in the acute phase. DSA on day two showed no aneurysm (Figure 3A), while magnetic resonance angiography on day 5 showed the reappearance of the aneurysm (Figure 3B). Moreover, DSA on day 14 showed an enlarged aneurysm (Figure 3C), and trapping with high-flow bypass was performed on day 15.

Craniotomy procedure

**Figure 4:**
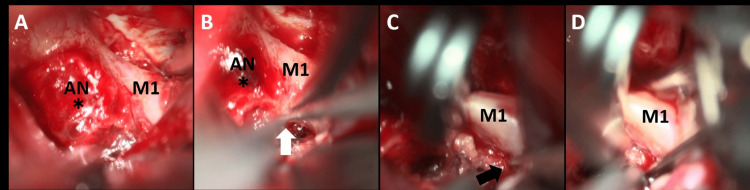
Direct surgery intraoperative findings (A) The pseudoaneurysm had the appearance of a very fragile blood clot (asterisk). (B-D) After confirming no obvious branching from the origin of the aneurysm, trapping was performed in the shortest section possible before (white arrow) and after (black arrow) the aneurysm AN: aneurysm; M1: middle cerebral artery M1 segment

A right frontotemporal craniotomy was performed, and after the anastomosis of the external carotid artery M2 using a left radial artery graft, the ICA bifurcation was temporarily blocked, and trapping was performed after confirming the presence of perforating branches in the M1 segment before and after the aneurysm neck in a flow reversal condition. After that, the ICA was opened and the treatment was completed. Intraoperatively, it was very difficult to open the Sylvian fissure due to brain swelling, and the pseudoaneurysm was firmly attached to the frontal lobe. The pseudoaneurysm resembled a very fragile blood clot (Figure 4A). Branching at the origin of the aneurysm was not observed, suggesting that it was a non-branching aneurysm (Figures 4B-4D).

Postoperative course

**Figure 5:**
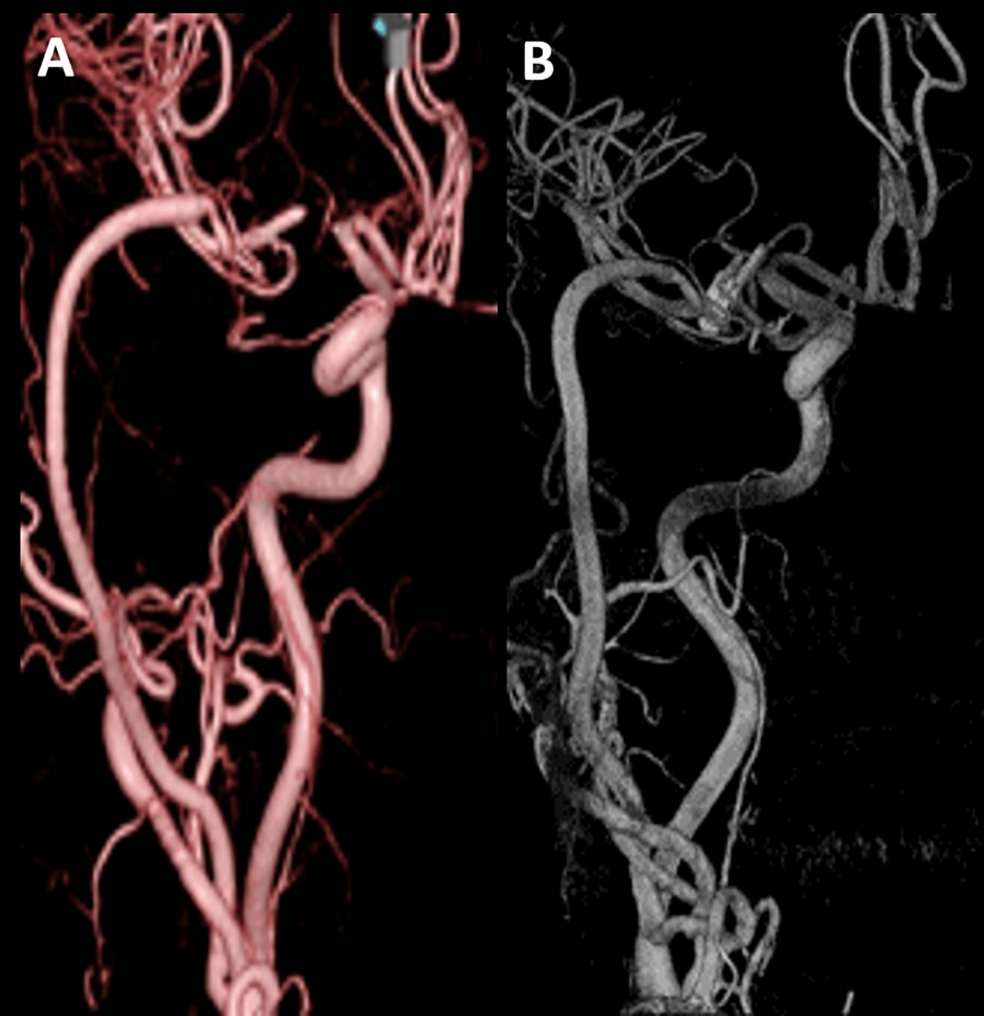
Postoperative DSA findings (A, B) DSA on postoperative day 28 and after one year showing good patency of bypass and no aneurysm DSA: digital subtraction angiography

The patient was administered a single antithrombotic drug the day after the operation, and MRI showed no new findings of cerebral infarction. DSA on postoperative good patency of the bypass was observed and no aneurysm was present on day 14 (Figure 5A). The patient was transferred to the hospital and had a Modified Rankin Scale score of 1 (rehabilitation for the disuse with long-term bed rest), although no neurological deficits were observed. DSA at one year after treatment showed no aneurysm recurrence and the bypass patency was excellent (Figure 5B).

## Discussion

This study presented two important findings. First, what appeared to be a non-branching saccular aneurysm in the M1 that was found to be the cause of SAH could have been a pseudoaneurysm. Second, trapping with high-flow bypass has been shown to be the safest and most reliable first-choice treatment procedure.

DSA showed that the aneurysm was not an obvious branching aneurysm but a continuous aneurysm with an irregular wall from a saccular aneurysm and that the entire aneurysm showed contrast delay and congestion, suggesting that the pathophysiology was different from that of a normal saccular aneurysm [4]. Non-branching aneurysms have been pathologically reported to be caused by pseudoaneurysms resulting from a small disruption of the internal elastic lamina. It has also been reported that half of the pseudoaneurysms formed in the main artery for anterior circulation other than the ICA that were found to be the cause of SAH had lacerations in the vessels that were considered normal on imaging, indicating that the aneurysm itself was formed by a thrombus [1].

In contrast, many studies have reported pseudoaneurysms forming at the rupture site of the saccular aneurysm rather than in the main artery wall, including pathological findings. In our case, although the aneurysm was at a non-branching site on CTA, it had a sac-like morphology with no wall irregularity, and hence it might be classified as a normal saccular aneurysm at first glance. However, the DSA results suggested that the aneurysm was a pseudoaneurysm forming over the rupture site of the saccular aneurysm. Thus, we determined that coil embolization was the feasible treatment for the saccular component. In fact, embolization was extremely difficult, and it is possible that some of the components we considered to be true saccular aneurysms may have been pseudoaneurysms formed by a thrombus. It is difficult to identify the extent of the rupture hole of the aneurysm and the boundary with the pseudoaneurysm on the images, which can be inferred from the distorted motion of the framing coil into the component we determined to be the true aneurysmal component. Furthermore, given that the entire aneurysm lost its visualization after coil retrieval, although many studies have reported cases of aneurysms losing their visualization for a short period of time due to various external factors [5], in our case, mechanical stimulation by temporary coil insertion and blood flow interruption with balloon assistance may have promoted the rapid thrombosis of the pseudoaneurysm. With regard to whether or not it was a branching aneurysm, it is difficult to confirm on DSA imaging due to the presence of intracranial hypertension and hematoma, and the possibility that it was a branching aneurysm of the M1 segment perforating branch cannot be denied. In any case, the imaging findings and clinical course were unique, and it was necessary to consider treatment strategies, considering that the main cause could be a pseudoaneurysm, even if it could be classified as a saccular aneurysm at first glance.

In our case, trapping with high-flow bypass was the safest and most reliable treatment procedure for a pseudoaneurysm in a non-branching M1 segment. In general, pseudoaneurysms require a more careful treatment method because they have walls formed by very fragile thrombi and bleed easily [1]. To date, an effective treatment approach for pseudoaneurysms has not been determined, and trapping with bypass has been considered the standard technique for an aneurysm of the anterior wall of the ICA. It has been reported that conventional neck clipping is associated with a high rate of fatal rebleeding during the procedure [6] and that coiling by endovascular procedure causes re-enlargement and rebleeding resulting in insufficient prophylactic effect [7]. In recent years, many studies have reported good outcomes of FD [8], but they are not currently available in Japan due to the lack of insurance coverage during the acute stage. On the other hand, for pseudoaneurysms other than ICA, the treatment strategy depends on the location of the aneurysm and the condition of the bleeding hole, but the same poor outcomes have been reported with conventional clipping and coiling for lesions in the proximal site of the MCA [9]. The choice of trapping with bypass in M1 lesions should be based on the risk of perforating branch infarction before and after M1 trapping as a complication. Therefore, it is important to maintain the retrograde blood flow to the maximum extent by using a high-flow bypass to maintain the blood flow in the perforating branch. In our case, we chose coil embolization as the acute treatment for a suspected pseudoaneurysm, with priority given to the early prevention of rebleeding, while keeping in mind the possibility of recurrence. We decided to perform frequent postoperative examinations and perform re-treatment in case of recurrence. Coil embolization into the pseudoaneurysm formed by the thrombus was difficult and abandoned early, and trapping with high-flow bypass was performed instead with good clinical outcomes. When the preoperative imaging findings indicate a pseudoaneurysm of the M1 segment, we believe that trapping with high-flow bypass might be the safer and more reliable procedure for this situation.

## Conclusions

Aneurysms at non-branching sites other than the ICA, which are observed in the preoperative examination of SAH, are often encountered. Based on the findings of this study, neurosurgeons should be mindful that even a small cerebral aneurysm that appears to be a saccular aneurysm at non-branching sites of M1 may be a very vulnerable pseudoaneurysm. We believe that trapping with high-flow bypass is the best treatment for pseudoaneurysm at non-branching sites of the MCA. Further case reports on the condition will be essential to determine the standard of care in the future.
